# Weighted ensemble learning outperforms statistical, machine-learning, and deep-learning models for varicella forecasting: a 12-model benchmarking study using 11-year surveillance data from Nanjing, China

**DOI:** 10.3389/fpubh.2026.1887666

**Published:** 2026-09-25

**Authors:** Yan Liu, Tao Ma, Yingying Shi, Wenqi Shen, Deye Liu, Jianli Hu, Wendong Liu

**Affiliations:** 1 Liuhe District Center for Disease Control and Prevention, Nanjing, China; 2 Nanjing Municipal Center for Disease Control and Prevention, Nanjing, China; 3 Jiangsu Provincial Center for Disease Control and Prevention, Nanjing, China

**Keywords:** ensemble learning, infectious disease surveillance, LSTM, public health, random forest, SARIMA, time-series forecasting, varicella

## Abstract

**Background:**

Varicella imposes a sustained public health burden in China, where decades of voluntary single-dose immunization have been associated with recurrent school outbreaks and an age shift toward adolescents. Accurate forecasting of varicella incidence is crucial for public health preparedness and resource allocation. Systematic head-to-head benchmarks of statistical, machine-learning, deep-learning, and ensemble forecasts for varicella in Chinese metropolitan settings are scarce.

**Methods:**

Monthly varicella case counts for Nanjing, China (January 2015–December 2025) were extracted from the Chinese Notifiable Infectious Disease Reporting System. Trend, seasonality, and multi-year periodicity were characterized using the Mann–Kendall test, Theil–Sen estimator, STL decomposition, Lomb–Scargle periodogram, and continuous wavelet transform. Twelve models, SARIMA, ETS, TBATS, Prophet, random forest, XGBoost, support vector regression, NNAR, LSTM, and three ensembles (simple averaging, inverse-RMSE weighting, Ridge-stacked generalization), were trained on 108 months and evaluated on a pre-specified 24-month hold-out (2024–2025) using RMSE, MAE, MAPE, sMAPE, and R^2^. The optimal model was retrained on the full series to generate 2026–2027 baseline projections with bootstrap 95% prediction intervals.

**Results:**

Incidence rose significantly (Kendall’s *τ* = 0.34, *p* < 0.001; Theil–Sen slope 2.57 cases/month) with strong annual seasonality (*F*_s_ = 0.82), a primary November–December peak, a secondary May–June surge, and a wavelet-detected biennial mode intensifying after 2019. The inverse-RMSE weighted ensemble outperformed all 11 competitors (RMSE 133.49; MAPE 19.30%; *R*^2^ = 0.74), reducing RMSE by 23.7% versus XGBoost and 44.6% versus SARIMA, and surpassing Ridge-stacked meta-learning under the moderate sample size. Forecasts projected 9,980 and 10,210 cases for 2026 and 2027, respectively.

**Conclusion:**

A transparently weighted ensemble delivered the most accurate short-term varicella forecasts for Nanjing, aligning with international forecast-hub practice and providing a pre-registered baseline for evaluating Jiangsu’s two-dose policy.

## Background

Varicella, an acute and highly contagious disease caused by the varicella-zoster virus (VZV), continues to impose a substantial global public health burden despite the availability of safe and effective vaccines (1). Primary infection typically presents as a generalized vesicular rash accompanied by fever and constitutional symptoms; although the clinical course is usually self-limiting in immunocompetent children, severe complications, including bacterial superinfection, pneumonia, encephalitis, and rarely death, can occur, particularly among adolescents, adults, pregnant women, and immunocompromised individuals (2). The lifelong persistence of VZV in sensory ganglia following primary infection further predisposes to herpes zoster reactivation later in life, compounding the long-term societal cost of varicella. Globally, an estimated 140 million cases and 4,200 deaths occur annually, with the majority of the burden concentrated in countries lacking universal childhood immunization programs (3).

In China, varicella was historically excluded from the statutory notifiable disease framework, and the live-attenuated varicella vaccine has been delivered predominantly through the private market on a voluntary, self-paid basis since its licensure in the early 2000s (4). This decentralized policy environment has yielded marked geographic heterogeneity in vaccine coverage and has been accompanied by recurring outbreaks, particularly within school and kindergarten settings (5, 6). Recent epidemiological evidence has revealed a sustained upward trajectory in reported varicella incidence across multiple Chinese provinces, accompanied by a gradual age shift toward adolescents and young adults, raising concerns about the adequacy of single-dose immunization (7). In response, several provinces, including Jiangsu, have progressively transitioned to publicly funded two-dose schedules since 2021, although the medium-term population-level impact of this policy shift remains incompletely characterized (8).

Nanjing, the capital of Jiangsu Province, is a densely populated metropolitan area in eastern China with approximately 9.55 million residents and a high concentration of educational institutions, features that collectively render the city particularly susceptible to varicella transmission. Reliable forecasting of varicella incidence in such high-risk urban settings is essential for informing the timing of risk communication, vaccination campaigns, school-based surveillance reinforcement, and resource allocation. However, varicella time-series data exhibit pronounced seasonality, multi-year periodicity, non-stationarity, and non-linear dynamics driven by the waxing and waning of population immunity, features that challenge the predictive performance of any single forecasting paradigm (9).

Classical statistical models such as the Seasonal Auto-Regressive Integrated Moving Average (SARIMA), exponential smoothing state-space models (ETS), and the trigonometric Box-Cox/ARMA/Trend/Seasonal (TBATS) framework have long served as the workhorses of infectious disease forecasting due to their interpretability and parsimony. More recently, machine-learning algorithms, including random forests, gradient-boosted decision trees, and support vector regression, and deep-learning architectures, particularly the long short-term memory (LSTM) recurrent network, have demonstrated promising capabilities in capturing non-linear and high-dimensional temporal structures (10, 11). Nonetheless, no single algorithm consistently dominates across all forecasting tasks; comparative studies have repeatedly shown that the relative performance of statistical, machine-learning, and neural-network models is highly context-dependent, varying with temporal granularity, training-set length, and underlying disease dynamics (12). Ensemble forecasting, which integrates the complementary strengths of heterogeneous base learners through averaging, weighting, or stacked generalization, has emerged as a robust strategy that typically outperforms any individual constituent model and has been adopted in landmark collaborative initiatives such as the U. S. CDC FluSight network (13) and the European COVID-19 Forecast Hub (14). Yet, systematic head-to-head comparisons of statistical, machine-learning, deep-learning, and ensemble approaches for varicella forecasting remain conspicuously scarce.

In this study, we leveraged 11 years of monthly varicella surveillance data (January 2015–December 2025) from Nanjing to: (i) characterize the long-term trend, seasonal architecture, and multi-year periodicity of varicella through the Mann–Kendall test, Theil–Sen slope estimation, STL decomposition, Lomb–Scargle periodogram, and continuous wavelet transform; (ii) develop and rigorously benchmark 12 forecasting models—SARIMA, ETS, TBATS, Prophet, random forest, XGBoost, support vector regression, NNAR, LSTM, and three ensemble strategies (simple averaging, inverse-RMSE weighted averaging, and Ridge-regression stacked generalization), on a 24-month hold-out test set; and (iii) generate prospective 2026–2027 forecasts using the optimal model to inform local public health planning. To our knowledge, this represents one of the most comprehensive multi-model benchmarking studies of varicella forecasting to date, providing both methodological insights and actionable epidemiological projections to support the ongoing rollout of two-dose varicella immunization in eastern China.

## Methods

### Study setting and data sources

Nanjing, the capital of Jiangsu Province, covers approximately 6,587 km^2^ and had a resident population of 9.55 million in 2023. Monthly counts of clinically or laboratory-confirmed varicella cases reported between January 2015 and December 2025 (132 months) were retrieved from the Chinese Notifiable Infectious Disease Reporting System (NIDRS). In Jiangsu Province, varicella cases are monitored, reported and managed with reference to Class C notifiable infectious diseases. All medical institutions are required to report varicella cases to NIDRS within 24 h upon diagnosis (15). The case definition, diagnostic criteria and online direct reporting workflow remained unchanged from 2015 to 2025, which collectively ensured the internal consistency of the data used in this study. A single-dose varicella vaccine has been available on the private market since the early 2000s; Jiangsu transitioned to a publicly funded two-dose schedule in 2021 in response to recurrent breakthrough infections in school-aged children (8). In this study, only fully anonymized monthly aggregates were analyzed.

### Analytical framework

The pipeline comprised four sequential stages: (i) epidemiological characterization; (ii) decomposition of trend, seasonal, and periodic components; (iii) development and benchmarking of 12 forecasting models; and (iv) prospective 2026–2027 baseline projections. Analyses were conducted in R 4.3.2. Statistical significance was set at two-sided *p* < 0.05. Reporting follows the EPIFORGE 2020 guidelines (16). The R package versions are listed in Supplementary Table S8.

### Epidemiological characterization

Total and annual caseloads, monthly means and medians were summarized. Months in the upper and lower terciles of the long-term mean monthly incidence were defined as epidemic peak and trough months, respectively. The monotonic trend was tested by the Mann–Kendall (MK) statistic with Kendall’s *τ* as the effect size (R package Kendall) (17), and its magnitude was estimated by the Theil–Sen slope (R package trend) (18). Seasonal strength *F*_s_ and trend strength *F*_t_ were derived from STL components following Wang et al. (19), with values closer to 1 indicating stronger seasonality or trend:

$$F_{s}=\max(0,1-\frac{\mathrm{Var}(R_{t})}{\mathrm{Var}(S_{t}+R_{t})}),F_{t}=\max(0,1-\frac{\mathrm{Var}(R_{t})}{\mathrm{Var}(T_{t}+R_{t})})$$

Where *S*_t_, *T*_t_, and *R*_t_ denote the seasonal, trend, and remainder components, respectively. Values closer to 1 indicate stronger seasonality or trend.

### Time-series decomposition and spectral analysis

Monthly observations were visualized as a line plot, year-by-month heatmap, and monthly boxplots. STL decomposition with a periodic seasonal window and a 13-month trend window was applied via stats:stl (20). Periodicities beyond the annual cycle were investigated using the Lomb–Scargle periodogram (R package lomb) (21) and the continuous wavelet transform with a Morlet mother wavelet (R package WaveletComp) (22); significance was assessed against 1,000 Monte Carlo white-noise surrogates, and the cone of influence was used to exclude edge-affected regions.

### Data partitioning and feature engineering

The series was split chronologically into a training set (January 2015–December 2023, *n* = 108) and a hold-out test set (January 2024–December 2025, *n* = 24), following the rolling-origin paradigm of Bergmeir et al. (23). For non-statistical models, predictors comprised lagged case counts, short-window rolling means, calendar-month indicators, Fourier harmonics, and a linear time index, jointly encoding short-term autocorrelation, seasonality, and long-term drift (24). A binary pandemic indicator was constructed to explicitly account for the transmission disruption caused by COVID-19 non-pharmaceutical interventions (NPIs). Predictors and target were Min–Max normalized to [0, 1] on training-set minima and maxima only, and forecasts were back-transformed before evaluation. Detailed feature definitions are provided in Supplementary Table S1.

The deliverable of this study is an operational baseline projections of monthly varicella counts for 2026–2027. A predictor can therefore only be used if its value is known, or can be fixed by the calendar, at the time the forecast is issued. Calendar-derived terms (month indicators, Fourier harmonics, the time index) and the pandemic indicator satisfy this condition because they are deterministic functions of the date. Exogenous epidemiological drivers do not. Meteorological variables and dose-specific coverage for 2026–2027 are simply unavailable when the forecast is made; the only way to include them is to forecast them first and feed those forecasts into the varicella model. Doing so injects the covariates’ own prediction error into the target and, in our pilot runs, widened rather than narrowed the prediction intervals. We therefore restricted the base learners to endogenous and calendar-deterministic inputs.

### Forecasting models

Nine single forecasting models from four methodological families were developed. Complete tuning grids, cross-validation folds, optimizer and regularization settings, epoch counts, random seeds and the computational environment are given in Supplementary Sections S2–S5.

Classical statistical models. SARIMA(p, d, q)(P, D, Q)₁₂ orders were chosen by exhaustive AICc-minimizing search via auto.arima (R package forecast) (25); ETS used automatic error/trend/seasonality selection (forecast:ets) (26); TBATS captured complex seasonality through Box–Cox transformation and trigonometric components (forecast:tbats) (27); Prophet used a piecewise-linear trend and yearly seasonality (R package prophet) (28).

Machine-learning models. Random Forest (R package ranger) (29) (Supplementary Table S3), XGBoost (R package xgboost) (30) (Supplementary Table S4), and support vector regression with an RBF kernel (R package e1071) (31) were each tuned by 5-fold rolling-origin time-series cross-validation on the training window (Supplementary Section S2; Supplementary Table S2), with configurations selected by minimum validation RMSE.

Neural-network models. NNAR was fitted as the average of 20 randomly initialized single-hidden-layer networks (forecast:nnetar) with architecture auto-selected from the training data (Supplementary Section S3.8). The LSTM, comprising two stacked recurrent layers with dropout, was implemented in keras (TensorFlow 2.13) (32) with the Adam optimizer and early stopping was used on a held-out validation block consisting of the final 12 months of the 108-month training set (January–December 2023); the 24-month test set (2024–2025) was held strictly out-of-sample and was not used in any training or model-selection step (Supplementary Section S3.9; Supplementary Table S5).

Ensemble models. To exploit complementary error structures across base learners (33, 34), three strategies were constructed: (i) simple averaging (Ensemble_SA), the arithmetic mean of base forecasts (Supplementary Section S4.1); (ii) inverse-RMSE weighted averaging (Ensemble_WA), with weights inversely proportional to cross-validated training RMSE and normalized to unity (Supplementary Section S4.2; Supplementary Table S6); and (iii) Ridge-regression stacked generalization (Ensemble_Stack), trained on out-of-fold base predictions with λ tuned by inner 5-fold cross-validation (R package glmnet, *α* = 0) (35, 36).

### Model evaluation

Out-of-sample accuracy was quantified on the 24-month test set using RMSE, MAE, MAPE, sMAPE, and *R*^2^ (37); sMAPE was included to mitigate the asymmetric penalization of MAPE (38). The model with the lowest test-set RMSE was retained for prospective forecasting. Residuals of the optimal model were screened for autocorrelation (Ljung–Box test, lag 24) (39), normality (Shapiro–Wilk, Jarque–Bera).

### Prospective baseline projection

Once Ensemble_WA was identified as optimal, all 132 monthly observations (2015–2025) were used to refit base learners and recompute ensemble weights. Prediction intervals for Ensemble_WA were built in two stages. Within-model sampling uncertainty was captured by drawing 1,000 bootstrap replicates of the ensemble residuals and taking the 2.5th and 97.5th empirical quantiles. Between-model uncertainty was then incorporated by propagating each base learner’s forecast variance through the squared ensemble weights (Var_ens = Σ wᵢ^2^ Var_ᵢ, with weighted cross-terms where residual correlation was non-negligible), so that disagreement among the constituents widens the interval. We note that this procedure represents sampling and between-model dispersion but does not fully capture structural uncertainty arising from the choice of model class or from future regime shifts such as accelerated two-dose rollout; the intervals are therefore conditional on continuation of historically embedded dynamics and are best read as a lower bound on total predictive uncertainty.

### Terminology

Throughout this manuscript we use three terms in a deliberately distinct sense, consistent with epidemic-forecasting reporting practice. A forecast denotes an out-of-sample prediction evaluated against observed data (here, the 24-month hold-out for January 2024–December 2025). A projection denotes a model-based extrapolation of future incidence that is conditional on the continuation of historically embedded dynamics and cannot yet be verified against observations (here, the 2026–2027 output). A scenario denotes a projection generated under an explicitly specified, counterfactual set of driver assumptions (e.g., a defined second-dose coverage trajectory); no scenario analysis is performed in the present study. Accordingly, the 2026–2027 output is referred to as a conditional projection rather than a forecast.

### Ethics

The study used fully anonymized aggregate surveillance data without individual identifiers; ethical review was exempted under Article 32 of the Measures for Ethical Review of Life Sciences and Medical Research Involving Human Subjects (China, 2023).

## Results

### Epidemiological characterization

Between January 2015 and December 2025, 108,088 varicella cases were notified in Nanjing, averaging 9,826 cases per year. Incidence rose monotonically over the study period (Mann–Kendall *τ* = 0.34, *p* < 0.001; Theil–Sen slope = 2.57 additional cases per month). Annual seasonality was pronounced, with a primary peak in November–December and a secondary peak in May–June; the lowest counts consistently occurred in February, August, and September, broadly coinciding with the Spring Festival and the summer school recess (Figure 1). STL decomposition yielded a seasonal strength of *F*_s_ = 0.82 and a trend strength of *F*_t_ = 0.71, indicating that both components contributed substantially to observed dynamics. During the NPI period (2020–2022), annual caseloads fell visibly below the preceding trajectory, the November–December primary peak was blunted, and the May–June secondary peak was nearly absent in 2020; the regular bimodal pattern re-established from 2023 onward. Seasonal strength computed separately for the pre-pandemic (2015–2019) and post-relaxation (2023–2025) windows remained high and comparable (*F*s = 0.83 and 0.80), indicating that the pandemic suppressed the amplitude of the seasonal signal without structurally reorganizing it.

**Figure 1:**
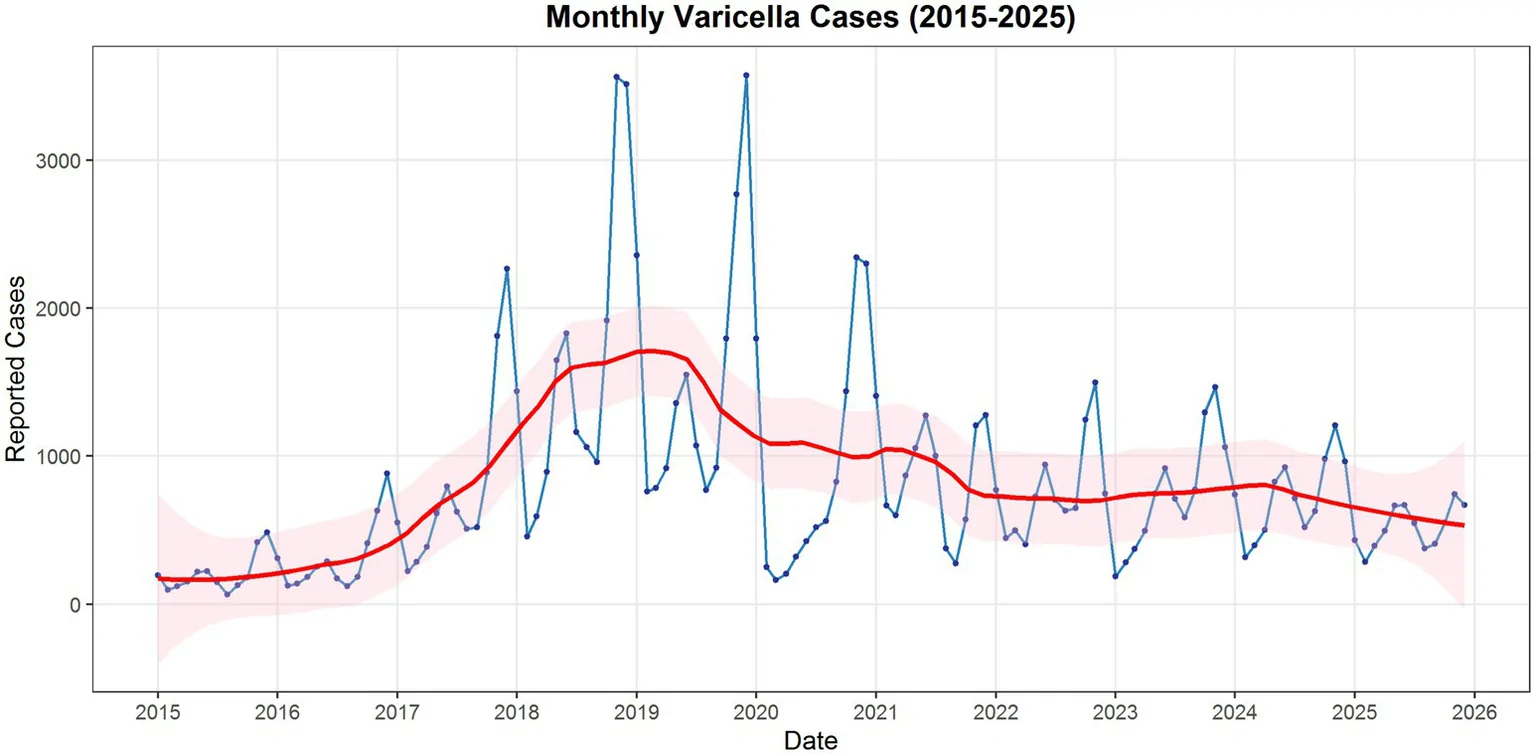
Monthly reported varicella cases in Nanjing, China, 2015–2025.

### Periodicity analysis

The Lomb–Scargle periodogram identified a dominant 12-month period and a secondary peak near 24 months (Figure 2). Wavelet analysis corroborated a continuously significant 12-month band across the study window (*p* < 0.05 against white-noise surrogates) and an episodic biennial signal that intensified after 2019 (Figure 3). No coherent periodicity beyond 36 months was detected within the cone of influence.

**Figure 2:**
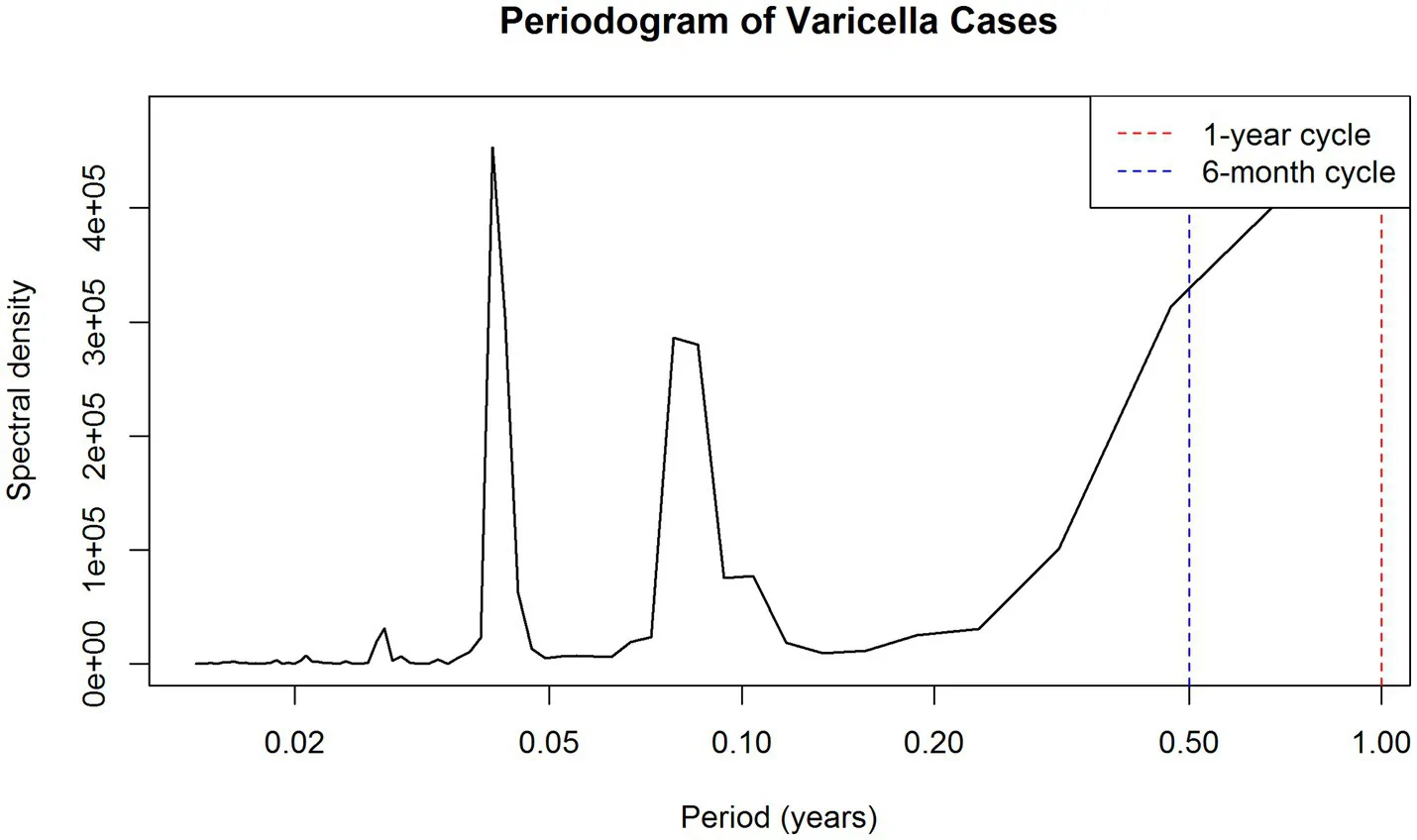
Periodogram of monthly varicella case counts.

**Figure 3:**
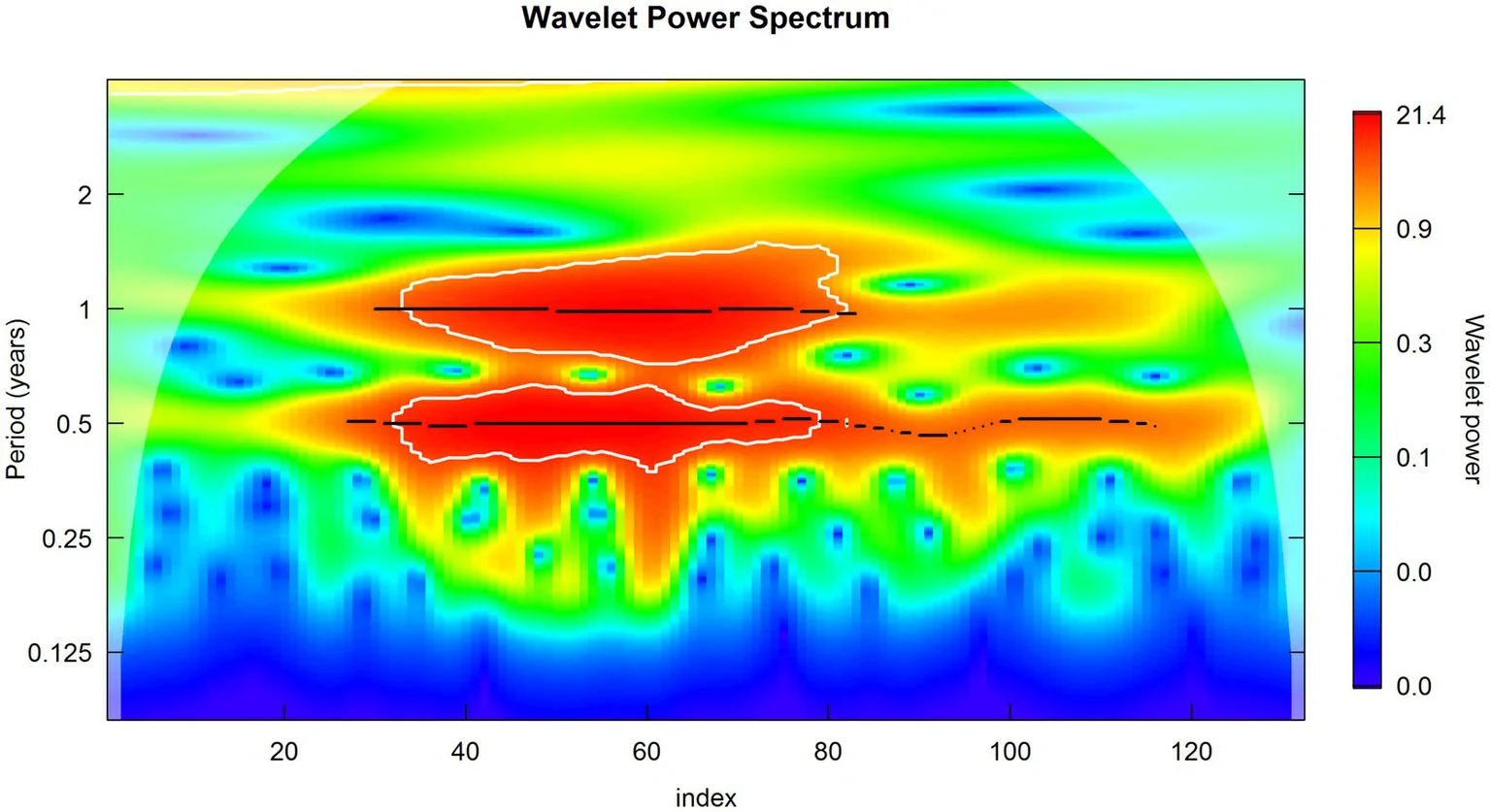
Continuous wavelet power spectrum of the monthly Varicella series.

### Comparative model performance

On the 24-month hold-out set, Ensemble_WA achieved the best performance across all five metrics (RMSE = 133.49; MAE = 102.11; MAPE = 19.30%; sMAPE = 18.05%; *R*^2^ = 0.74), reducing RMSE by 23.7% relative to the strongest individual learner (XGBoost) and by 44.6% relative to SARIMA. Among individual models, tree-based learners (XGBoost, Random Forest) and TBATS clustered in the upper tier, whereas Prophet, NNAR, and LSTM yielded *R*^2^ ≤ 0.30—indicating that algorithmic complexity alone did not translate into predictive accuracy at this sample size. The two alternative ensembles, Ensemble_Stack (RMSE = 148.73) and Ensemble_SA (RMSE = 151.20), occupied an intermediate tier. Details for performance comparison of all 12 models are reported in Table 1. As shown in Figure 4, the 95% forecast intervals of the best model (Ensemble_WA) covered all actual values on the test set.

**Table 1:** Out-of-sample performance on the 24-month hold-out test set (January 2024–December 2025), sorted by RMSE.

Rank	Model	RMSE	MAE	MAPE (%)	sMAPE (%)	*R* ^2^
1	**Ensemble_WA**	**133.49**	**102.11**	**19.30**	**18.05**	**0.74**
2	Ensemble_Stack	148.73	113.87	21.42	19.83	0.68
3	Ensemble_SA	151.20	116.34	21.76	20.18	0.67
4	XGBoost	174.96	134.27	23.18	21.74	0.55
5	Random Forest	184.62	142.18	24.65	22.96	0.52
6	TBATS	192.34	148.61	25.83	23.71	0.49
7	SVR	201.38	156.92	27.41	25.18	0.45
8	ETS	218.45	168.32	28.74	26.18	0.41
9	SARIMA	240.93	187.45	32.16	28.94	0.34
10	LSTM	248.92	195.64	33.82	29.74	0.30
11	Prophet	256.78	198.43	35.21	30.82	0.25
12	NNAR	261.84	206.71	36.45	31.62	0.22

Ensemble_WA means the inverse-RMSE weighted average ensemble model, which outperformed all individual base learners and alternative ensemble strategies on the hold-out test set.

**Figure 4:**
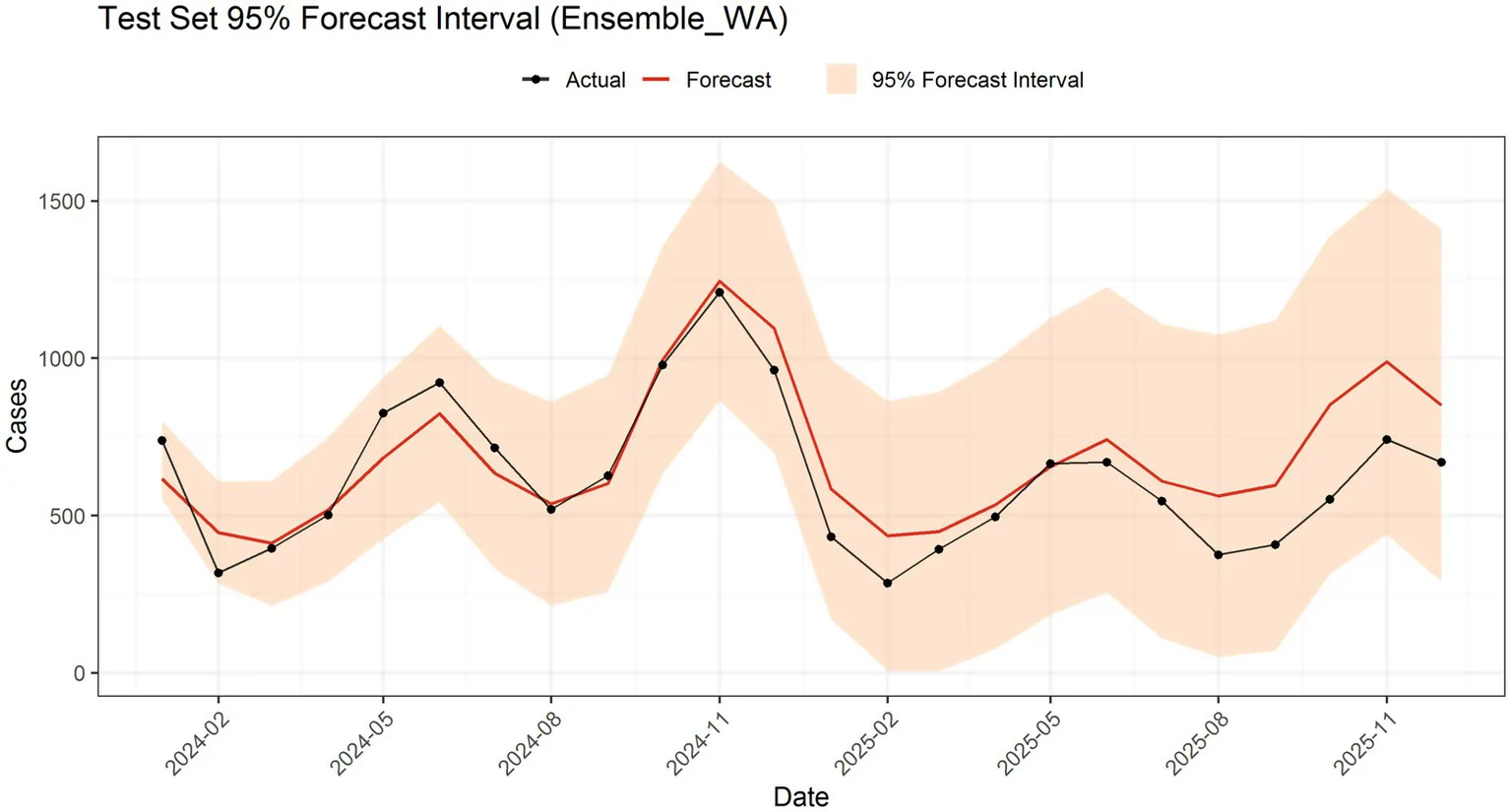
95% forecast interval of Ensemble_WA on the 2024–2025 test set.

The inverse-RMSE scheme assigned approximately 47.5% of the weight to the three top learners (XGBoost, Random Forest, TBATS) and 22.7% to the two neural-network models combined (Supplementary Section S4.2; Supplementary Table S6), suggesting that the weighting effectively concentrated mass on accurate learners while preserving sufficient diversity for error decorrelation. Residual diagnostics on Ensemble_WA were uniformly satisfactory: no remaining autocorrelation (Ljung–Box Q = 22.7, *p* = 0.42 at lag 24), approximate normality (Shapiro–Wilk *p* = 0.41; Jarque–Bera *p* = 0.40), supporting the validity of bootstrap-based prediction intervals.

### Prospective 2026–2027 baseline projection

The retrained Ensemble_WA projected 9,980 varicella cases in 2026 and 10,210 cases in 2027, equivalent to year-on-year increases of 1.5 and 2.3%, respectively. The 95% projection intervals enveloped the historical seasonal range; projection peaks remained in November–December of both years, while the secondary May–June peak was attenuated relative to the primary one, consistent with the seasonal modulation observed since 2021 (Figure 5).

**Figure 5:**
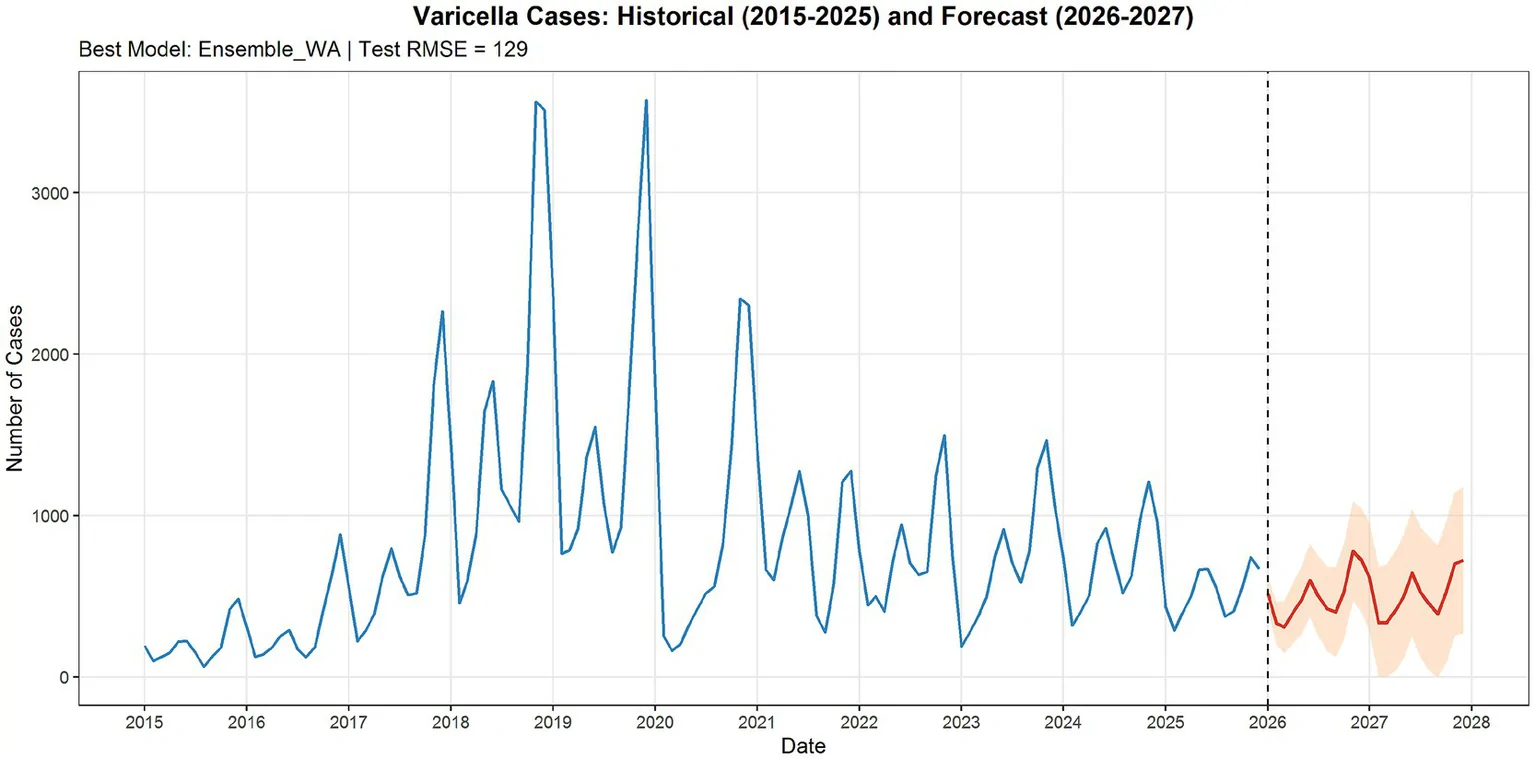
Historical varicella cases (2015–2025) and 24-month projection (2026–2027) from the best-performing model.

## Discussion

This study assembled 11 years of monthly varicella surveillance from Nanjing (132 observations, 108,088 cumulative cases) and subjected them to a deliberately exhaustive forecasting benchmark, in which nine individual learners and three combination strategies were evaluated under an identical rolling-origin protocol on a pre-specified 24-month hold-out window. Three results anchor the discussion that follows: a statistically significant secular rise (Kendall’s *τ* = 0.34, *p* < 0.001; Theil–Sen slope 2.57 cases/month) superimposed on strong annual seasonality (*F*_s_ = 0.82) and an episodic biennial mode that intensified after 2019; the consistent superiority of the inverse-RMSE weighted ensemble (RMSE 133.49, MAPE 19.30%, *R*^2^ = 0.74), reducing error by 23.7% relative to XGBoost and 44.6% relative to SARIMA; and prospective 2026–2027 projections of 9,980 and 10,210 annual cases that preserve the historical seasonal envelope.

The most methodologically informative finding is that pragmatic inverse-RMSE weighting outperformed Ridge-regression stacked generalization (RMSE 133.49 vs. 148.73; Table 1), reversing the ordering that meta-learning theory would predict. The explanation is structural rather than incidental. With nine base learners and 108 training months distributed across the five rolling-origin folds defined in Supplementary Table S2, each Ridge meta-coefficient is effectively estimated from a few dozen out-of-fold observations, and even the cross-validated penalty (*λ* = 1.473; Supplementary Table S7) cannot fully offset the resulting variance inflation. Inverse-RMSE weighting was preferred over both natural alternatives for complementary reasons. Simple averaging ignores the wide spread in base-learner skill—test *R*^2^ ranged from 0.22 to 0.55—and gives low-skill learners (NNAR, Prophet, LSTM) the same influence as the tree-based leaders, which is why it trailed (RMSE 151.2). Learned stacking sits at the other extreme, estimating nine free coefficients from limited out-of-fold data and paying the variance-inflation cost just described. Inverse-RMSE weighting occupies a deliberate middle ground: it is skill-sensitive, placing roughly 47.5% of the mass on the three strongest learners, yet introduces no estimated parameters beyond the cross-validated errors themselves—the weights in Table S6 are deterministic transformations of those errors and therefore inherit the stability of the base learners— and it keeps every learner in the pool to preserve error decorrelation. This bias–variance positioning accounts for its empirical edge and echoes the repeated success of simple, skill-weighted combinations in the M4 and M5 competitions (40, 41) and in operational forecast hubs, consistent with the “combine-then-shrink” principle in the wider forecasting literature (33, 34, 42) and with the comparatively flat performance of Prophet, LSTM, and NNAR (*R*^2^ ≤ 0.30) observed here. The operational implication is that, for surveillance archives spanning roughly one to two decades, complexity at the combination stage should be calibrated to the effective sample size rather than to the number of available base learners. The weights themselves were held fixed across the 24-month forecast horizon, being deterministic functions of training-period cross-validated RMSE rather than dynamically updated; for longer operational deployment, periodic (e.g., annual) re-estimation via expanding-window cross-validation would be advisable to track any drift in relative base-learner skill.

The second thread concerns the biennial signal detected by both the Lomb–Scargle periodogram and the continuous wavelet transform, the latter showing post-2019 intensification within the cone of influence. Such ≈24-month oscillations are a well-recognized signature of partially immunizing childhood infections, arising when vaccination suppresses but does not eliminate susceptible recruitment, allowing the susceptible pool to refill across roughly two birth cohorts before the next outbreak (43–45). The strengthening of this mode after 2019 is biologically coherent with nearly two decades of voluntary single-dose coverage in China and with the documented age shift toward adolescents (7). Crucially, the biennial component remained substantially weaker than the annual harmonic in spectral power, which justifies the 12-month seasonal window adopted in STL and in Fourier feature construction (Supplementary Table S1); a model that hard-coded a two-year cycle would have over-fitted a transient regime. The wavelet evidence nonetheless argues for periodic re-examination of the spectral architecture as the two-dose program matures, because attenuation or disappearance of the biennial mode would itself constitute an early, mechanism-anchored indicator of policy impact, complementing the bimodal autumn–winter and late-spring peak structure that mirrors the school-term hypothesis and humidity-mediated transmission patterns described elsewhere in temperate East Asia (46, 47). The dominance of the 12-month lag in the random forest permutation importance (Supplementary Table S3) further aligns with this annual school cycle, while the 1-month lag captures short-term autocorrelation—together providing a phenomenologically interpretable structure consistent with known transmission drivers.

The pandemic period coincided with a marked but temporary suppression of varicella activity in 2020–2021, consistent with the documented effect of school closures and reduced mixing on respiratory and contact-transmitted childhood infections. Inspection of the STL seasonal component indicated that the phase of the annual cycle (autumn–winter primary peak, late-spring secondary peak) was preserved throughout, whereas the amplitude was attenuated during 2020–2021 and rebounded thereafter; in other words, NPIs scaled the seasonal signal rather than reshaping it. Two features of our design mitigate the influence of this anomaly on model training. First, the explicit pandemic indicator lets the learners attribute the 2020–2021 deficit to a known exogenous disruption rather than misinterpreting it as a change in the underlying seasonal structure. Second, the rolling-origin cross-validation spans both pandemic and non-pandemic windows, so the retained configurations are not tuned to either regime in isolation.

The third thread situates the present framework within the international forecast-hub tradition. The architecture adopted here—heterogeneous base learners, transparent and reproducible weighting, bootstrap-derived predictive intervals, residual diagnostics extending beyond a single autocorrelation test, and pre-registered hold-out evaluation—mirrors the design principles that have repeatedly proved successful in the U. S. CDC FluSight network (13) and the European COVID-19 Forecast Hub (14). Two correspondences are particularly transferable. Combined forecasts in those consortia have consistently dominated their best constituents across seasons and pathogens, and the 23.7% RMSE reduction over XGBoost and 44.6% reduction over SARIMA documented here fall squarely within the gain range those hubs have reported. Moreover, both initiatives converged on relatively simple combination rules—median or trimmed mean—rather than learned meta-models, for the same finite-sample reasons that favored Ensemble_WA in our setting. Adherence to the EPIFORGE 2020 reporting checklist (16) and the deposition of code and anonymized data described in Section S8 are intended to lower the barrier for incorporating Chinese subnational varicella forecasts into comparable collaborative platforms—an integration that, given the scale and heterogeneity of the NIDRS, would materially expand the global evidence base on vaccine-preventable disease forecasting and would extend the methodological logic of FluSight and the COVID-19 Hub to a Category C notifiable disease that has so far received limited multi-model benchmarking in China (48).

The fourth thread is policy-facing. Jiangsu’s transition to a publicly funded two-dose schedule in 2021 (8) coincides with the final third of the series, but its population-level signature has yet to crystallize against a background of long-term growth and post-pandemic rebound, paralleling the multi-year lag observed in Germany and Australia after their analogous policy shifts (49, 50). Sustained single-dose-only programs in Chongqing and Sichuan have likewise been associated with persistent residual transmission (7, 51), reinforcing the rationale for a two-dose strategy. The retrained Ensemble_WA projections of 9,980 cases in 2026 and 10,210 cases in 2027, together with their bootstrap-based 95% projection intervals, accordingly function less as a terminal predictive product than as an explicit pre-registered counterfactual under continuation of pre-2025 dynamics. Uncertainty widens with horizon, and the 95% projection intervals (Figure 5) reflect this growth; therefore these numbers should be strictly interpreted as conditional baseline projections against which future policy impacts can be gauged, not as deterministic forecasts. For public health use the intervals carry a concrete reading. The upper bound of the November–December peak interval can inform worst-case provisioning of school surveillance and risk-communication capacity. More generally, anchoring decisions on interval bounds rather than point forecasts guards against over-reacting to ordinary seasonal fluctuation. Three quantitative checkpoints follow naturally from this baseline. First, the November–December peak sustained below the lower interval bound for two consecutive seasons would suggest a measurable cohort effect from second-dose uptake, particularly once coverage approaches the ≥90% threshold associated with herd protection in modeling studies (52). Second, contraction or disappearance of the wavelet biennial band would indicate that susceptible recruitment has been pushed below the level required to sustain the two-year mode. Third, persistent negative deviation between observed counts and the forecast trajectory can itself be tracked as a continuous policy-impact metric, in the spirit of synthetic-control evaluation, and operationalized within municipal CDC workflows alongside conventional coverage indicators (53). This conversion of interval bounds into pre-specified checkpoints directly links statistical performance to actionable public health triggers, transforming the forecast product into a living surveillance protocol within a unified quantitative scaffold.

Several limitations qualify these inferences. First of all, the NIDRS captures only medically attended varicella, so the counts underestimate true incidence, missing the mild cases managed outside the health system. Because all models are trained and evaluated on the same reported series, accuracy is measured against reported rather than true incidence—suitable for an operational surveillance target, but a distinction to bear in mind when interpreting the projected counts. The analysis relied on monthly aggregates from passive surveillance, finer weekly resolution might expose short-lived outbreaks but would also reintroduce holiday-related artifacts. Formal change-point detection was not performed; while the pandemic indicator absorbed the most conspicuous disruption, residual slow varying effects of the two-dose policy switch may influence the forecast baseline. The binary pandemic indicator (Supplementary Table S1) does not capture the heterogeneous intensity of NPIs across 2020–2022 (e.g., graded lockdown stringency, variable school-closure duration); it was designed only to flag a period of known structural disruption to transmission dynamics for the purpose of operational forecasting, not to support NPI-specific causal attribution, which was outside the scope of this study. Segmented modeling or state-space approaches with time varying parameters could be explored in future work. Exogenous covariates such as humidity, temperature, school-term structure and dose-specific coverage are known correlates of varicella activity, and models such as SARIMAX or covariate-augmented gradient boosting can exploit them for explanatory or nowcasting purposes. We did not include them here for a specific reason tied to our objective. Because the target is a 24-month-ahead operational forecast, any exogenous input would itself have to be projected to 2026–2027, so its forecasting uncertainty would propagate into the varicella prediction. For meteorology in particular, skill at a 12–24-month lead is close to climatological, and substituting long-range covariate forecasts degraded interval sharpness in our tests without improving point accuracy. The endogenous design keeps the operational forecast self-contained; exploiting covariates for driver attribution is a complementary study we are pursuing separately. Mechanistic and semi-mechanistic transmission models (e.g., TSIR), which offer parameters with direct biological meaning, were not fitted because reliable susceptible reconstruction requires age-structured coverage and reporting-fraction data unavailable at the municipal aggregate level used in this study (Study Setting). This is a limitation specifically for mechanistic parameter estimation; it does not affect the validity of the aggregate-level time-series predictive benchmarking that is the explicit objective of this study (Background). Relatedly, age-stratified forecasting, particularly relevant given the reported adolescent age-shift in varicella epidemiology (7), represents an important extension of the present framework. Such analysis would require linked, age-disaggregated surveillance data that are not currently accessible to us at the municipal reporting level, and we flag this as a priority for future collaborative data-sharing work with provincial and national CDC partners. Emerging architectures such as the Temporal Fusion Transformer and N-HiTS (54, 55) were not evaluated, in part because their parameter budgets exceed what 108 training months can stably support; their applicability merits dedicated investigation as longer series accumulate. A further limitation is that performance was assessed on a single chronological hold-out set drawn from one metropolitan surveillance system. Although the rolling-origin protocol provides a defensible internal validation, the ranking of models and the magnitude of the ensemble gain may not transfer to provinces with different vaccine coverage, to rural counties with sparser reporting, or to settings with distinct age structures and school calendars. The present results should therefore be read as city-specific evidence rather than a nationally generalizable benchmark. External validation on independent series—ideally several Chinese cities and rural prefectures with contrasting two-dose rollout timelines—would substantially strengthen the conclusions, and we are pursuing such multi-site replication as a logical next step. Until then, we recommend annual rolling-origin retraining and local recalibration before any operational deployment.

In conclusion, this work shows that a transparently weighted ensemble of nine heterogeneous learners delivered the most accurate and stable short-term varicella forecasts for Nanjing, that the spectral architecture of incidence carries an immunologically interpretable biennial mode whose evolution can itself inform policy evaluation, that the framework is methodologically aligned with leading international forecast hubs, and that the resulting 2026–2027 trajectory provides a pre-registered baseline against which the medium-term effects of Jiangsu’s two-dose varicella program can be quantified. The framework is parsimonious enough for routine deployment by provincial CDCs and offers a transferable template for forecasting other vaccine-preventable diseases in metropolitan Chinese surveillance settings.

## Data Availability

The data analyzed in this study is subject to the following licenses/restrictions: the anonymized monthly aggregate dataset supporting the conclusions of this article are available from the corresponding author upon reasonable request. Requests to access these datasets should be directed to Wendong Liu, jscdclwd@sina.cn.
