# Supplementary material for: Weighted ensemble learning outperforms statistical, machine-learning, and deep-learning models for varicella forecasting: a 12-model benchmarking study using 11-year surveillance data from Nanjing, China
Source: Front Public Health. 2026 Sep 25;14:1887666. doi: 10.3389/fpubh.2026.1887666 (PMC13649673; doi:10.3389/fpubh.2026.1887666)
Supplement: Supplementary file 1 [file Data_Sheet_1.PDF]

Supplementary Materials

Weighted ensemble learning outperforms statistical, machine-learning, and deep-learning models for varicella forecasting: a 12-model benchmarking study using 11-year surveillance data from Nanjing, China

Contents

S1. Engineered predictors for non-statistical models

S2. Rolling-origin cross-validation scheme

S3. Fitted specifications and tuned hyperparameters of individual models (S3.1–S3.9)

S4. Ensemble strategies: weights and meta-learner coefficients (S4.1–S4.3)

S5. Software environment and reproducibility

S1. Engineered predictors for non-statistical models

This section expands the brief description in the Methods (Data partitioning and feature engineering). The four classical statistical models (SARIMA, ETS, TBATS, Prophet) operated directly on the univariate monthly series; machine-learning and neural-network models required an explicit predictor matrix. All predictors and the target were Min–Max scaled to [0, 1] using training-set minima and maxima; forecasts were back-transformed before evaluation.

Table S1. Engineered predictors used by Random Forest, XGBoost, SVR, NNAR, and LSTM

Feature group	Definition	Purpose
Lagged values	y_{t−1}, y_{t−2}, y_{t−3}, y_{t−6},	Short-term autocorrelation;

	y_{t−12}	annual recurrence
Rolling means	3- and 6-month moving averages	Smoothed medium-term level
Calendar month	One-hot indicators (Jan–Dec)	Discrete seasonal effects
Fourier terms	sin(2πkt/12), cos(2πkt/12), k = 1, 2	Smooth periodic component
Time index	t = 1, …, 132	Long-term linear drift
Pandemic indicator	Binary variable: 1 for Jan 2020–Dec 2022, 0 otherwise	Absorbs NPI-related level shift; protects seasonal/AR structure

S2. Rolling-origin cross-validation scheme

This section details the CV protocol referenced in the Methods (Individual forecasting models). Five expanding-window folds preserved temporal ordering; the configuration minimizing average validation RMSE was retained and refitted on the full 108-month training set before application to the test set.

Table S2. Rolling-origin cross-validation folds

Fold	Training months	Validation months
1	1–48	49–60
2	1–60	61–72
3	1–72	73–84
4	1–84	85–96
5	1–96	97–108

S3. Fitted specifications and tuned hyperparameters

This section reports the technical details intentionally omitted from the Methods. Test-set performance is consolidated in Section S5 to avoid duplication.

S3.1 SARIMA. Augmented Dickey − Fuller did not reject non-stationarity ($P = 0.21$); KPSS rejected level stationarity ($P = 0.01$). After one non-seasonal and one seasonal difference ($d = 1$, $D = 1$), both tests indicated stationarity (ADF $P < 0.01$; KPSS $P > 0.10$). Exhaustive AICc-minimizing search selected SARIMA$(0, 1, 4)(0, 1, 1)_{12}$, with AICc = 1373.21 and BIC = 1387.58. The residual series is a white noise process (Box-Ljung test: $Q = 0.0569$, $p = 0.811$).

S3.2 ETS

Automatic AICc-based selection across 30 candidates returned ETS(A, N, A) (additive errors, no trend, additive seasonality), with smoothing parameters $\alpha = 0.675$ and $\gamma = 0.0001$ (AICc = 1643.287). The very small γ is consistent with the highly stable seasonal pattern ($F_s = 0.82$).

S3.3 TBATS

Auto-selection yielded a Box–Cox parameter $\lambda = 0.628$ (near square-root), no damping, ARMA(0, 0) errors, and a 12-month seasonality represented by K = 5 Fourier pairs: TBATS(0.026, {0,0}, –, {<12,5>}).

S3.4 Prophet

Piecewise-linear trend with 25 default changepoint candidates, additive seasonality, yearly Fourier order = 10, changepoint prior scale = 0.05, seasonality prior scale = 10. Holiday regressors were not used because monthly aggregation already absorbs holiday-related variation; weekly and daily seasonalities were disabled.

S3.5 Random Forest

The selected configuration is summarized in Table S4. Permutation importance was dominated by the 1-month lag (38.4%), followed by the 12-month lag (19.7%) and month (12.1%), confirming the centrality of annual recurrence.

Table S3. Tuned hyperparameters for Random Forest

Hyperparameter	Search space	Selected
num.trees	300, 500, 1000	500
mtry	2–8	4
min.node.size	5, 10, 20	5

S3.6 XGBoost

Regularisation for XGBoost was provided by shrinkage (eta = 0.05), row and column subsampling (0.8/0.8), a minimum child weight of 3 and a split-loss penalty (gamma = 0.1), alongside early stopping at round 286.

Table S4. Tuned hyperparameters for XGBoost

Hyperparameter	Search space	Selected
eta	0.01, 0.05, 0.1	0.05
max_depth	3, 5, 7	5
subsample	0.7, 0.8, 1.0	0.8
colsample_bytree	0.7, 0.8, 1.0	0.8
min_child_weight	1, 3, 5	3
gamma	0, 0.1, 0.3	0.1
nrounds	up to 1000	286 (early-stopped)

S3.7 Support Vector Regression

RBF kernel, ε-insensitive loss; Tuning grid: $C \in \{1, 10, 100\}$, $\gamma \in \{0.01, 0.05, 0.1\}$, $\varepsilon \in \{0.05, 0.1, 0.2\}$; selected $C = 10$, $\gamma = 0.05$, $\varepsilon = 0.1$. Tuned configuration: $C = 10$, $\gamma = 0.05$, $\varepsilon = 0.1$.

S3.8 NNAR

Auto-selected architecture: $\text{NNAR}(4, 1, 3)_{12}$ — four non-seasonal lags, one seasonal lag, three hidden neurons (logistic activation), weight decay enabled, 20 random initializations averaged. Wider hidden layers were tested but degraded validation performance, consistent with overfitting at this sample size.

S3.9 LSTM

In contrast to the brief specification in the Methods, the following provides the complete LSTM architecture and training schedule (Table S6). Inputs were 12-month sliding windows of the scaled series. Two stacked LSTM layers were each followed by dropout (rate = 0.2). Adam (lr = 0.001) minimized MSE; early stopping (patience = 20) on the final 12 training months halted training at epoch 156 (training loss 0.0089; validation loss 0.0142). Random seeds were fixed at 42 in R, NumPy, and TensorFlow.

Table S5. LSTM architecture and training configuration

Component	Specification
Input	Samples=96, timesteps=1, features=24
LSTM layer 1	64 units, return_sequences = TRUE
Dropout 1	0.2
LSTM layer 2	32 units, return_sequences = FALSE

Dropout 2	0.2
Output	Dense(1), linear activation
Trainable parameters	18,201
Loss / Optimizer	MSE / Adam (lr = 0.001)
Batch size / Epochs	16 / 200 (patience = 20)

Regularization comprised two dropout layers (rate 0.2) and early stopping (patience 20); no explicit L2 weight penalty was applied, consistent with the small training sample.

S4. Ensemble strategies

This section provides the numerical weights and coefficients underlying the ensemble strategies defined in the Methods.

S4.1 Simple averaging (Ensemble_SA)

Equal-weighted mean of the nine base forecasts, included as a non-informative benchmark.

S4.2 Inverse-RMSE weighted averaging (Ensemble_WA)

Weights were normalized inverses of cross-validated training RMSE (Table S7).

Table S6. Inverse-RMSE weights for Ensemble_WA

Model	CV RMSE	Weight w_i
XGBoost	168.43	0.1672
Random Forest	178.92	0.1574
TBATS	187.51	0.1502
SVR	198.74	0.1417
ETS	211.65	0.1331
SARIMA	232.18	0.1213
LSTM	241.07	0.1168
Prophet	248.92	0.1131
NNAR	256.13	0.1100

S4.3 Ridge-regression stacked generalization (Ensemble_Stack)

Out-of-fold predictions served as inputs to a Ridge meta-learner (glmnet, $\alpha = 0$); λ was tuned over a logarithmic grid (10^{-3} to 10^2) with the optimum at $\lambda = 1.473$ (Table S8). The relative underperformance of stacking compared with weighted averaging (Section S5) is consistent with the well-documented unreliability of meta-learning under high parameter-to-observation ratios.

Table S7. Ridge meta-learner coefficients

Term	β	Standardized β
Intercept	18.42	—
XGBoost	0.231	0.219
Random Forest	0.187	0.176
TBATS	0.156	0.151
SVR	0.121	0.117
ETS	0.108	0.103
SARIMA	0.094	0.089
Prophet	0.062	0.058
LSTM	0.058	0.053
NNAR	0.041	0.038

S5. Software environment and reproducibility

All analyses were run under R 4.3.2, LSTM training used TensorFlow 2.13 through the R keras interface with Python 3.10. Random seeds were fixed at 42 in R.

Table S8. Principal software dependencies

R Package	Version	Role
forecast	8.21	SARIMA, ETS, TBATS, NNAR
prophet	1.0	Prophet
ranger	0.15.1	Random Forest
xgboost	1.7.5.1	XGBoost
e1071	1.7-13	SVR
glmnet	4.1-8	Ridge stacking
keras	2.13.0	LSTM interface

WaveletComp	1.1	Wavelet analysis
lomb	2.1.2	Lomb–Scargle periodogram
