# Supplementary material for: Weighted ensemble learning outperforms statistical, machine-learning, and deep-learning models for varicella forecasting: a 12-model benchmarking study using 11-year surveillance data from Nanjing, China
Source: Front Public Health. 2026 Sep 25;14:1887666. doi: 10.3389/fpubh.2026.1887666 (PMC13649673; doi:10.3389/fpubh.2026.1887666)
Supplement: Supplementary file 2 [file Data_Sheet_2.PDF]

**Supplementary Materials**

# Weighted ensemble learning outperforms statistical, machine-learning, and deep-learning models for varicella forecasting: a 12-model benchmarking study using 11-year surveillance data from Nanjing, China

## Contents


S1. Engineered predictors for non-statistical models

S2. Rolling-origin cross-validation scheme

S3. Fitted specifications and tuned hyperparameters of individual models (S3.1–S3.9)

S4. Ensemble strategies: weights and meta-learner coefficients (S4.1–S4.3)

S5. Software environment and reproducibility


## S1. Engineered predictors for non-statistical models

This section expands the brief description in the Methods (Data partitioning and feature engineering). The four classical statistical models (SARIMA, ETS, TBATS, Prophet) operated directly on the univariate monthly series; machine-learning and neural-network models required an explicit predictor matrix. All predictors and the target were Min–Max scaled to [0, 1] using training-set minima and maxima; forecasts were back-transformed before evaluation.

Table S1. Engineered predictors used by Random Forest, XGBoost, SVR, NNAR, and LSTM

| Feature group | Definition | Purpose |
|---|---|---|
| Lagged values | y_{t−1}, y_{t−2}, y_{t−3}, y_{t−6}, | Short-term autocorrelation; |

| | | |
|---|---|---|
| | y_{t−12} | annual recurrence |
| Rolling means | 3- and 6-month moving averages | Smoothed medium-term level |
| Calendar month | One-hot indicators (Jan–Dec) | Discrete seasonal effects |
| Fourier terms | sin(2πkt/12), cos(2πkt/12), k = 1, 2 | Smooth periodic component |
| Time index | t = 1, …, 132 | Long-term linear drift |
| Pandemic indicator | Binary variable: 1 for Jan 2020–Dec 2022, 0 otherwise | Absorbs NPI-related level shift; protects seasonal/AR structure |

## S2. Rolling-origin cross-validation scheme

This section details the CV protocol referenced in the Methods (Individual forecasting models). Five expanding-window folds preserved temporal ordering; the configuration minimizing average validation RMSE was retained and refitted on the full 108-month training set before application to the test set.

Table S2. Rolling-origin cross-validation folds

| Fold | Training months | Validation months |
|---|---|---|
| 1 | 1–48 | 49–60 |
| 2 | 1–60 | 61–72 |
| 3 | 1–72 | 73–84 |
| 4 | 1–84 | 85–96 |
| 5 | 1–96 | 97–108 |

## S3. Fitted specifications and tuned hyperparameters

This section reports the technical details intentionally omitted from the Methods. Test-set performance is consolidated in Section S5 to avoid duplication.

S3.1 SARIMA. Augmented Dickey－Fuller did not reject non-stationarity ($P = 0.21$); KPSS rejected level stationarity ($P = 0.01$). After one non-seasonal and one seasonal difference ($d = 1$, $D = 1$), both tests indicated stationarity (ADF $P < 0.01$; KPSS $P > 0.10$). Exhaustive AICc-minimizing search selected SARIMA$(0, 1, 4)(0, 1, 1)_{12}$, with AICc = 1373.21 and BIC = 1387.58. The residual series is a white noise process (Box-Ljung test: $Q = 0.0569$, $p = 0.811$).

### S3.2 ETS

Automatic AICc-based selection across 30 candidates returned ETS(A, N, A) (additive errors, no trend, additive seasonality), with smoothing parameters $\alpha = 0.675$ and $\gamma = 0.0001$ (AICc = 1643.287). The very small $\gamma$ is consistent with the highly stable seasonal pattern ($F_s = 0.82$).

### S3.3 TBATS

Auto-selection yielded a Box–Cox parameter $\lambda = 0.628$ (near square-root), no damping, ARMA(0, 0) errors, and a 12-month seasonality represented by K = 5 Fourier pairs: TBATS(0.026, {0,0}, –, {<12,5>}).

### S3.4 Prophet

Piecewise-linear trend with 25 default changepoint candidates, additive seasonality, yearly Fourier order = 10, changepoint prior scale = 0.05, seasonality prior scale = 10. Holiday regressors were not used because monthly aggregation already absorbs holiday-related variation; weekly and daily seasonalities were disabled.

### S3.5 Random Forest

The selected configuration is summarized in Table S4. Permutation importance was dominated by the 1-month lag (38.4%), followed by the 12-month lag (19.7%) and month (12.1%), confirming the centrality of annual recurrence.

**Table S3.** Tuned hyperparameters for Random Forest

| Hyperparameter | Search space | Selected |
|---|---|---|
| num.trees | 300, 500, 1000 | 500 |
| mtry | 2–8 | 4 |
| min.node.size | 5, 10, 20 | 5 |

### S3.6 XGBoost

Regularisation for XGBoost was provided by shrinkage (eta = 0.05), row and column subsampling (0.8/0.8), a minimum child weight of 3 and a split-loss penalty (gamma = 0.1), alongside early stopping at round 286.

**Table S4.** Tuned hyperparameters for XGBoost

| Hyperparameter | Search space | Selected |
|---|---|---|
| eta | 0.01, 0.05, 0.1 | 0.05 |
| max_depth | 3, 5, 7 | 5 |
| subsample | 0.7, 0.8, 1.0 | 0.8 |
| colsample_bytree | 0.7, 0.8, 1.0 | 0.8 |
| min_child_weight | 1, 3, 5 | 3 |
| gamma | 0, 0.1, 0.3 | 0.1 |
| nrounds | up to 1000 | 286 (early-stopped) |

### S3.7 Support Vector Regression

RBF kernel, ε-insensitive loss; Tuning grid: $C \in \{1, 10, 100\}$, $\gamma \in \{0.01, 0.05, 0.1\}$, $\varepsilon \in \{0.05, 0.1, 0.2\}$; selected $C = 10$, $\gamma = 0.05$, $\varepsilon = 0.1$. Tuned configuration: $C = 10$, $\gamma = 0.05$, $\varepsilon = 0.1$.

### S3.8 NNAR

Auto-selected architecture: $NNAR(4, 1, 3)_{12}$ — four non-seasonal lags, one seasonal lag, three hidden neurons (logistic activation), weight decay enabled, 20 random initializations averaged. Wider hidden layers were tested but degraded validation performance, consistent with overfitting at this sample size.

### S3.9 LSTM

In contrast to the brief specification in the Methods, the following provides the complete LSTM architecture and training schedule (Table S6). Inputs were 12-month sliding windows of the scaled series. Two stacked LSTM layers were each followed by dropout (rate = 0.2). Adam (lr = 0.001) minimized MSE; early stopping (patience = 20) on the final 12 training months halted training at epoch 156 (training loss 0.0089; validation loss 0.0142). Random seeds were fixed at 42 in R, NumPy, and TensorFlow.

**Table S5.** LSTM architecture and training configuration

| Component | Specification |
|---|---|
| Input | Samples=96, timesteps=1, features=24 |
| LSTM layer 1 | 64 units, return_sequences = TRUE |
| Dropout 1 | 0.2 |
| LSTM layer 2 | 32 units, return_sequences = FALSE |

| | |
|---|---|
| Dropout 2 | 0.2 |
| Output | Dense(1), linear activation |
| Trainable parameters | 18,201 |
| Loss / Optimizer | MSE / Adam (lr = 0.001) |
| Batch size / Epochs | 16 / 200 (patience = 20) |

Regularization comprised two dropout layers (rate 0.2) and early stopping (patience 20); no explicit L2 weight penalty was applied, consistent with the small training sample.

## S4. Ensemble strategies

This section provides the numerical weights and coefficients underlying the ensemble strategies defined in the Methods.

### S4.1 Simple averaging (Ensemble_SA)

Equal-weighted mean of the nine base forecasts, included as a non-informative benchmark.

### S4.2 Inverse-RMSE weighted averaging (Ensemble_WA)

Weights were normalized inverses of cross-validated training RMSE (Table S7).

**Table S6.** Inverse-RMSE weights for Ensemble_WA

| Model | CV RMSE | Weight w_i |
|---|---|---|
| XGBoost | 168.43 | 0.1672 |
| Random Forest | 178.92 | 0.1574 |
| TBATS | 187.51 | 0.1502 |
| SVR | 198.74 | 0.1417 |
| ETS | 211.65 | 0.1331 |
| SARIMA | 232.18 | 0.1213 |
| LSTM | 241.07 | 0.1168 |
| Prophet | 248.92 | 0.1131 |
| NNAR | 256.13 | 0.1100 |

### S4.3 Ridge-regression stacked generalization (Ensemble_Stack)

Out-of-fold predictions served as inputs to a Ridge meta-learner (glmnet, $\alpha = 0$); $\lambda$ was tuned over a logarithmic grid ($10^{-3}$ to $10^{2}$) with the optimum at $\lambda = 1.473$ (Table S8). The relative underperformance of stacking compared with weighted averaging (Section S5) is consistent with the well-documented unreliability of meta-learning under high parameter-to-observation ratios.

**Table S7.** Ridge meta-learner coefficients

| Term | β | Standardized β |
|---|---|---|
| Intercept | 18.42 | — |
| XGBoost | 0.231 | 0.219 |
| Random Forest | 0.187 | 0.176 |
| TBATS | 0.156 | 0.151 |
| SVR | 0.121 | 0.117 |
| ETS | 0.108 | 0.103 |
| SARIMA | 0.094 | 0.089 |
| Prophet | 0.062 | 0.058 |
| LSTM | 0.058 | 0.053 |
| NNAR | 0.041 | 0.038 |

## S5. Software environment and reproducibility

All analyses were run under R 4.3.2, LSTM training used TensorFlow 2.13 through the R keras interface with Python 3.10. Random seeds were fixed at 42 in R.

**Table S8.** Principal software dependencies

| R Package | Version | Role |
|---|---|---|
| forecast | 8.21 | SARIMA, ETS, TBATS, NNAR |
| prophet | 1.0 | Prophet |
| ranger | 0.15.1 | Random Forest |
| xgboost | 1.7.5.1 | XGBoost |
| e1071 | 1.7-13 | SVR |
| glmnet | 4.1-8 | Ridge stacking |
| keras | 2.13.0 | LSTM interface |

| | | |
|---|---|---|
| WaveletComp | 1.1 | Wavelet analysis |
| lomb | 2.1.2 | Lomb–Scargle periodogram |
